# Graphene Oxide Enhanced Cisplatin Cytotoxic Effect in Glioblastoma and Cervical Cancer

**DOI:** 10.3390/molecules28176253

**Published:** 2023-08-25

**Authors:** Kacper Kregielewski, Wiktoria Fraczek, Marta Grodzik

**Affiliations:** 1 Faculty of Biology and Biotechnology, Warsaw University of Life Sciences, 02-787 Warsaw, Poland; 2 Department of Nanobiotechnology, Institute of Biology, Warsaw University of Life Sciences, 02-787 Warsaw, Poland

**Keywords:** cisplatin, graphene oxide, cancer, glioblastoma, cervical cancer

## Abstract

Graphene oxide (GO) is an oxidized derivative of graphene. So far, GO has mostly been studied as a drug delivery method rather than a standalone drug for treating cancers like glioblastoma or cervical cancer. However, we propose a promising new approach—using GO as a sensitizer for cisplatin chemotherapy. Here, we analyze the effects of triple GO pretreatment, followed by cisplatin treatment, on cancerous cell lines U87 and HeLa, as well as the noncancerous cell line HS-5, through morphology analysis, viability assay, flow cytometry, and LDH release assay. The viability assay results showed that GO treatment made U87 and HeLa cells more responsive to cisplatin, leading to a significant reduction in cell viability to 40% and 72%, respectively, without affecting HS-5 cells viability, while the Annexin V/Propidium iodine assay showed that GO pretreatment did not cause a change in live cells in all three examined cell lines, while GO-pretreated HeLa cells treated with cisplatin showed significant decrease around two times compared to cells treated with cisplatin standalone. The U87 cell line showed a significant increase in LDH release, approximately 2.5 times higher than non-GO-pretreated cells. However, GO pretreatment did not result in LDH release in noncancerous HS-5 cells. It appears that this phenomenon underlays GO’s ability to puncture the cell membrane of cancerous cells depending on its surface properties without harming noncancerous cells.

## 1. Introduction

In 2020, cancers were responsible for around 10 million deaths worldwide [1]. Glioblastoma is a primary malignant brain tumor, the most common brain tumor diagnosed in adults worldwide [2]. Glioblastoma is one of the deadliest human cancers with only 3 to 5% of patients surviving more than 3 years after the diagnosis [3]. Due to the presence of a highly selective blood–brain barrier, therapy with chemotherapeutics is often not successful [4]. One of the confirmed glioblastoma risk factors is exposure to ionizing radiation, typically seen years after exposure and associated with the treatment of other cancers. Some environmental risk factors, such as smoking, vinyl chloride, or pesticide exposure, have been associated with glioblastoma occurrence. Increased risk of glioblastoma is associated with some genetic disorders such as Li-Fraumeni syndrome, tuberous sclerosis, and retinoblastoma [5]. Cervical cancer is the third most common cancer type diagnosed in women [1], and while in well-developed countries it does not cause many deaths, it is still a common cause of death in less developed countries due to the lack and high prices of screening tests [6]. The most important risk factor in cervical cancer is human papillomavirus (HPV) with HPV-16 and HPV-18 being the leading types. Other cervical cancer risk factors include smoking, early sexual initiation, and low socioeconomic level [7]. Usage of oral contraceptive pills for more than 5 years can double the risk of cervical cancer occurrence [8]. Cervical cancer and glioblastoma were selected for the experiment due to the frequency of diagnosis and mortality. Simultaneously, both cancers have different cisplatin sensitivity, location in the human body, and anatomical limitations to therapy applications. Using two completely different cancers allows us to test the pretreatment scheme on a broad range of cancer cases.

Cancer therapy is time-consuming, is associated with multiple side effects, and still often does not have the intended effects. While cancers differ in many ways such as origin or location in the human body, very often one therapeutic agent, such as cisplatin, is used to treat multiple cancer types.

Graphene has a two-dimensional structure made up of sp^2^ carbons arranged in a hexagonal honeycomb-shaped structure [9]. Graphene oxide (GO) is an oxidized derivative of graphene with a structure similar to that of its precursor. GO has additional carbon atoms in sp^3^ hybridization and functional groups such as carboxyl, hydroxyl, carbonyl, or epoxy groups. Due to the presence of additional groups, GO was successfully functionalized using DNA, proteins, or peptides [10]. GO cytotoxicity and genotoxicity depend on the oxidative state, the presence of particular functional groups such as C=O, and the oxygen content of GO [11], which depend on the way GO was prepared. For example, GO synthesized using chemical methods has some structural differences from GO prepared by a thermal method [12]. Previous studies on GO toxicity showed that it can reduce HeLa cells’ viability, trigger reactive oxygen species (ROS) release, and reduce glioblastoma U87 cells’ viability [13].

Cisplatin has been a popular cytostatic drug in cancer therapy since 1978 [14]. The action mechanism of cisplatin is based on its ability to form crosslinks between DNA, resulting in DNA damage and inhibition of proliferation abilities. The most commonly observed crosslinking appears to be between cisplatin and N7 atoms of guanine [15]. Cisplatin has an ability to form adducts: monoadducts and double adducts. Monoadduct formation results in a genotoxic effect while double adduct formation results in cytotoxic properties of cisplatin [16]. Crosslinks prevent cell repair which leads to apoptosis [17]. In addition to apoptosis induction, cisplatin can also lead to cell necrosis [18]. Cell proliferation is affected by cisplatin treatment, resulting in initial arrest in the S phase, with further arrest in the G2/M phase [16]. Another drawback of cisplatin is the ability of cancer cells to develop cellular resistance, by altering the DNA repair or by modifying cisplatin uptake mechanisms. Cellular resistance to cisplatin can be also caused by mitochondria alterations, autophagy dysfunctions, apoptosis inhibition, tumor microenvironment changes, or cisplatin inactivation inside the cancer cells [19]. There is no data on cisplatin’s lethal dose for humans, but for rats, it is between 8 to 45 mg/kg depending on the administration route [20]. For human cancer cell lines, the minimal IC50 value for cisplatin is 0.177 μM in the Ewings sarcoma EW-3 line, while the maximal IC50 is 10,373 μM in the pancreas PANC-04-03 cancer cell line [21] Cisplatin therapy is widely used in lung, prostate, ovaries, cervix, and breast cancers [16]. It was previously described that cisplatin was used in combined therapy clinical tests, with other drugs such as capecitabine [22] and paclitaxel [23].

Cisplatin is a non-selective drug, which means that except for therapeutical properties, it also causes many side effects to the patients such as nephrotoxicity, neurotoxicity, and myelosuppression [5,24].

Previous studies showed that GO can attach to the cell surface and cause cell membrane damage by extracting single phospholipids, creating pores, and causing lipid peroxidation and membrane leakage that enlarge in a dose- and time-dependent manner [25,26]. We hypothesize that this phenomenon can ease and increase the uptake of drugs into the cell. This study aims to determine whether triple-time repeated low-dose GO administration can sensitize cancer cells to cisplatin treatment.

## 2. Results and Discussion

### X-ray Photoelectron Spectroscopy (XPS) of GO

**Figure 1:**
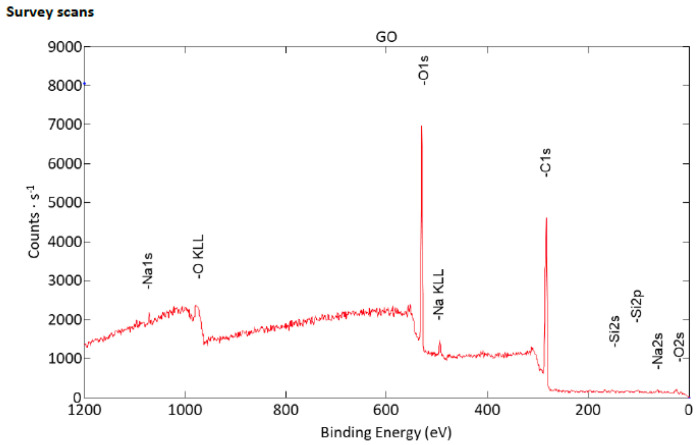
XPS result of GO sample: survey spectra scan.

**Figure 2:**
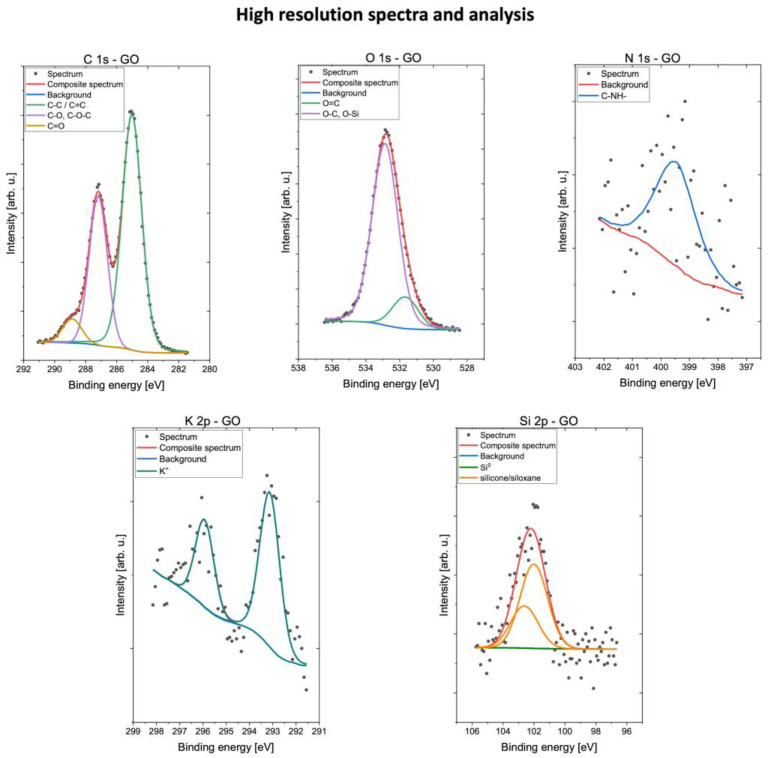
XPS result of GO sample: high-resolution spectra.

Results of the XPS survey of the GO sample are shown in Figure 1. Carbon and oxygen were the most common components of the surface composition at 96.8 atomic %. Some residues were also found with low concentrations of nitrogen atoms found at 399.8 eV that due to the noisy signal could be erroneous, and spectra signals at 102.3 eV indicated the presence of silicone- and/or siloxane-type compounds or SiO_2_ [27,28]. Data also showed the signal characteristic for the presence of potassium ions at 293.2 eV, which can be residue from the potassium permanganate used in the modified hummers method that was used to produce the GO in the experiment [27,29,30]. Detailed results of surface composition determined by fitting XPS data are presented in Table 1. High-resolution spectra of GO are shown in Figure 2.

**Figure 3:**
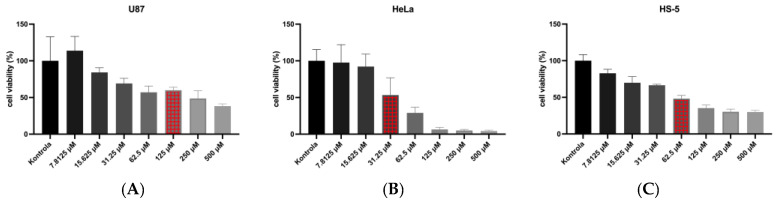
Dose-response of cisplatin concentrations on U87 (**A**); HeLa (**B**); and HS-5 (**C**) cells, using fluorescence measurement. The red bar indicates the concentration that was used for the subsequent phase of the experiment.

To determine the dose-response of cisplatin for a further experiment, an AlamarBlue assay was performed. The results are shown in Figure 3. Based on the AlamarBlue test, a 125 μM concentration of cisplatin was selected for the U87 cell line, 31.25 μM for HeLa, and 62.5 μM for HS-5 cells.

**Figure 4:**
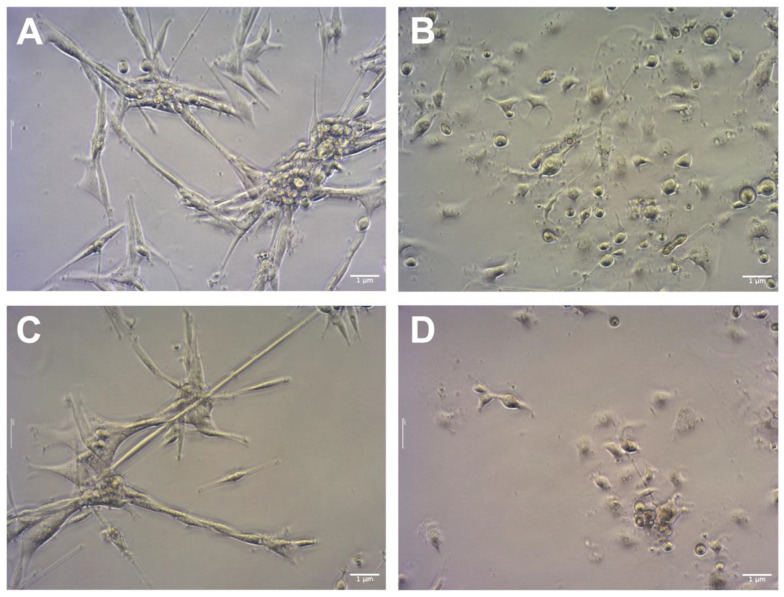
Comparison of U87 cells’ morphology: control (**A**); cells treated with cisplatin (**B**); cells pretreated with GO (**C**); cells pretreated with GO and treated with cisplatin (**D**). Scale bar 1 μm; 10× objective.

**Figure 5:**
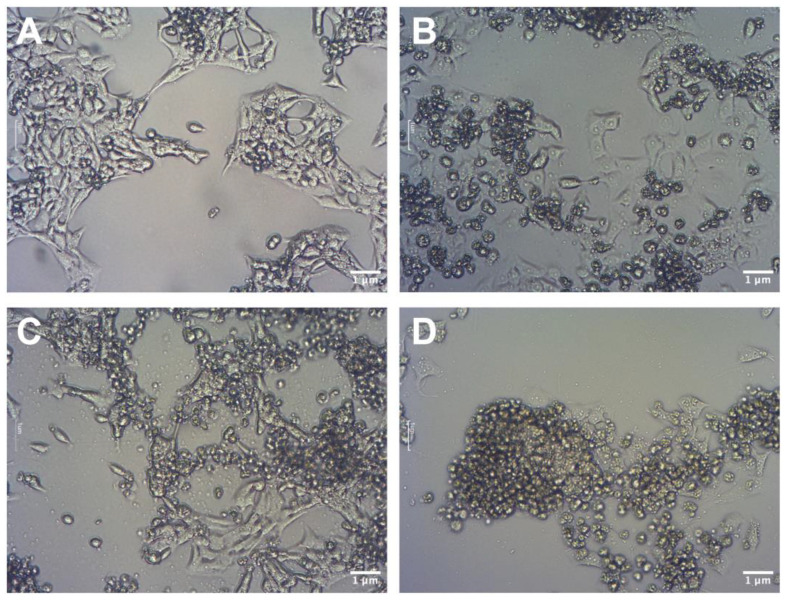
Comparison of HeLa cells’ morphology: control (**A**); cells treated with cisplatin (**B**); cells pretreated with GO (**C**); cells pretreated with GO and treated with cisplatin (**D**). Scale bar 1 μm; 10× objective.

**Figure 6:**
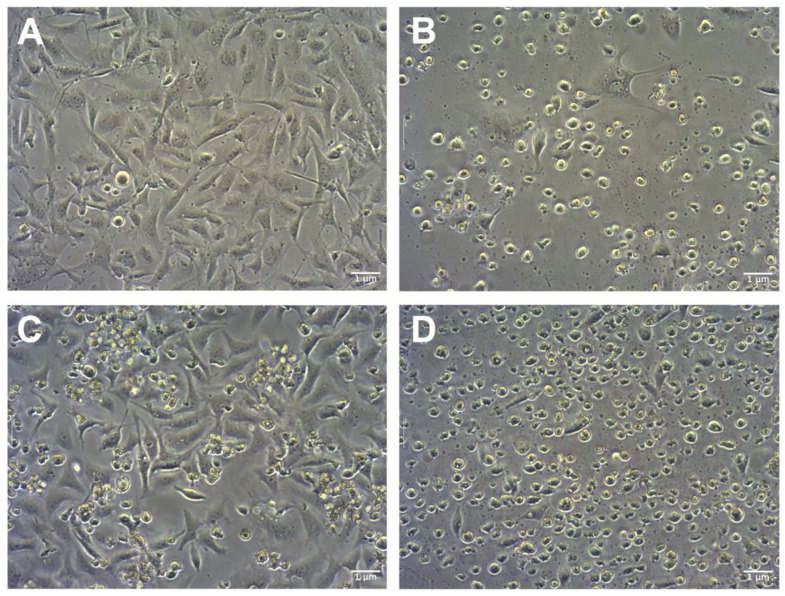
Comparison of HS-5 cells’ morphology: control (**A**); cells treated with cisplatin (**B**); cells pretreated with GO (**C**); cells pretreated with GO and treated with cisplatin (**D**). Scale bar 1 μm; 10× objective.

The use of GO has the purpose of sensitizing cancer cells for further treatment with cytostatic drugs such as cisplatin. A pretreatment scheme was used in studies regarding colon cancer cells, which were sequentially pretreated with 2-Oxohexyl isothiocyanate followed by 5-FU administration [31]. Because previous studies showed that GO in higher concentrations can be toxic [13] it was decided to pretreat cells with three low doses of GO (1 μg/mL). The influence of GO, cisplatin, and GO + cisplatin on the morphology of U87 (Figure 4), HeLa (Figure 5), and HS-5 (Figure 6) cell lines was evaluated using light microscopy. For the U87 control group (Figure 4A), characteristic cell shapes were observed. Cells presented a tendency to grow in close contact, leading to the formation of spheroids. GO-treated U87 cells (Figure 4C) did not show any difference in cell morphology, but the number of cells was lower than that of the control group. In the cisplatin-treated group (Figure 4B), cells of altered morphology and dead cells were observed. The group of U87 pretreated with GO and treated with cisplatin (Figure 4D) presented a decrease in cells of typical morphology. Rounded dead cells were observed, similar looking to those described by Kutwin et al. [32]. The nontreated HeLa control group (Figure 5A) presented characteristic cell line morphology. In the cisplatin-treated group (Figure 5B) and the pretreated with GO and treated with cisplatin group (Figure 5D), numerous dead cells were observed. In the pretreated with GO and treated with cisplatin HeLa cells, the number of dead cells was higher than in cells treated solely with cisplatin (Figure 5B). The number of live HeLa cells pretreated with GO (Figure 5C) was similar to the number of cells in the control group (Figure 5A), but also showed slightly more dead cells. In the HS-5 control group (Figure 6A), characteristic HS-5 cells were observed with visible nuclei in different cell cycle stages. HS-5 cells treated with GO (Figure 6C) also presented characteristic cell morphology but also showed circular dead cells that were not attached to the culture flask. In cisplatin-treated HS-5 (Figure 6B) cells, few cells of typical morphology and multiple dead cells were observed. HS-5 cells pretreated with GO and treated with cisplatin (Figure 6D) presented more live cells than in the cisplatin-treated group (Figure 6B). Significantly more dead cells were observed there as well.

**Figure 7:**
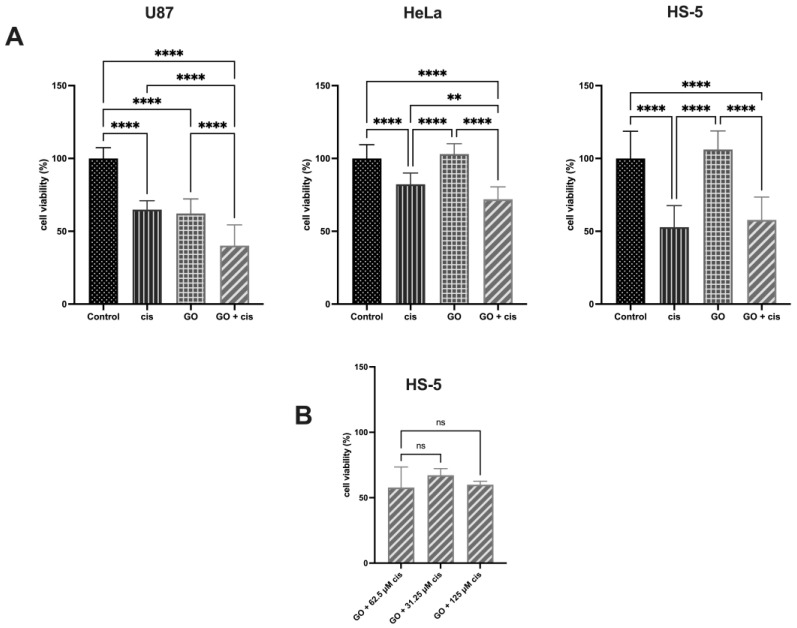
(**A**) Comparison of the viability of U87, HeLa, and HS-5 cells pretreated with GO, treated with cisplatin, and pretreated with GO that was given cisplatin after GO treatment. Control cells not treated with GO or cisplatin were used. (**B**) Comparison of different cisplatin concentrations on GO-treated HS-5 cells. Cisplatin concentrations were the same as used for the U87 (125 μM) and HeLa (31.25 μM) cell lines. Fluorescence measurement was used to read the results. Four asterisks (****) mean *p*-value ≤ 0.0001, two asterisks (**) mean *p*-value ≤ 0.01 and ns means result not statistically significant. Abbreviations: cis, cells treated with cisplatin in specific concentration for each line; GO, cells pretreated with 1 μg/mL GO; GO + cis, cells pretreated with 1 μg/mL GO and given cisplatin in specific concentrations for each line after GO treatment.

The AlamarBlue test was performed to determine the influence of cisplatin on cells pretreated with GO, with results presented in Figure 7A. Cisplatin treatment caused U87 cells to decrease to around 65% while cisplatin treatment of GO-pretreated U87 cells resulted in a decrease of around 40% viability compared to the control cells. U87 GO-pretreated cells were found to have a decrease in viability similar to the cisplatin-treated group. These results differ from those reported by Jaworski et al. who showed that five times higher GO concentrations do not affect U87 viability [13]. This may be due to the extended incubation time with GO, as it is known that GO can cause damage to the cells in a time- and concentration-dependent manner [26]. For the HeLa line, cisplatin caused a decrease in the viability of non-GO-pretreated cells to around 82%, while cisplatin treatment caused HeLa GO-pretreated cells’ viability to significantly decrease to 72%. In the HS-5 cell line, cisplatin caused a decrease in non-GO-pretreated cells to around 53%, while the viability of GO-pretreated cells that received cisplatin decreased to 58%, with no significant difference. Additionally, a comparison of cisplatin concentrations used for cancerous cell lines and the effect on noncancerous HS-5 lines was performed, and the results are shown in Figure 7B. Analysis showed that there were no differences in HS-5 cell viability regardless of cisplatin concentration on previously GO-pretreated cells, suggesting that additional GO pretreatment of noncancerous cells would not cause additional damage. The above-stated could mean that GO treatment on noncancerous cells does not appear to increase the dose-dependent manner of cisplatin toxicity. No effect on GO-treated noncancerous HS-5 cell viability was previously described by Jaworski et al. [33].

**Figure 8:**
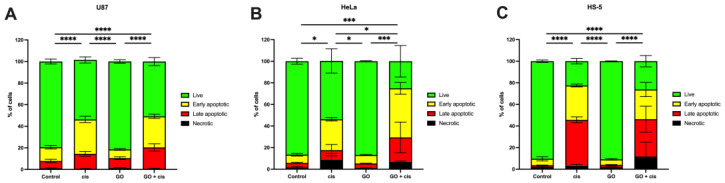
Comparison of the cell state of U87 (**A**); HeLa (**B**); and HS-5 cells (**C**) pretreated with GO, treated with cisplatin, and pretreated with GO that were given cisplatin after GO treatment. For control cells, those not treated with GO or cisplatin were used. Change in % of live cells is indicated by the bar. Four asterisks (****) mean *p*-value ≤ 0.0001, three asterisks (***) mean *p*-value ≤ 0.001 and one asterisk (*) means *p*-value ≤ 0.05. Abbreviations: cis, cells treated with cisplatin in a specific concentration for each line; GO, cells pretreated with 1 μg/mL GO; GO + cis, cells pretreated with 1 μg/mL GO that were given cisplatin in a specific concentration for each line after GO treatment.

Both GO and cisplatin are known to cause apoptosis in many ways; the most common is reactive oxygen generation (ROS) dependent [34,35], and thus Annexin V/Propidium iodine apoptosis flow cytometry assay was performed. The results of the flow cytometry are presented in Figure 8. Table containing results of each assay can be found in the Appendix A. Apoptosis assay for U87 cells showed that GO pretreatment followed by cisplatin administration caused a significant decrease in live cells, to around 58%, compared to the control group. There was no significant difference between the groups treated with GO + cisplatin and the group treated with cisplatin. GO treatment followed by cisplatin significantly reduced live cells to around 58% compared to cells treated with GO (~81.5%). In both cisplatin-treated groups, late and early apoptotic cells were found. For HeLa cells, a significant decrease in live cell % was found between cells treated with just cisplatin and GO-pretreated cells that were given cisplatin—the percentages of live cells were around 54% and 25%, respectively. GO and cisplatin-treated cells had the lowest count of live cells than any other group. No significant change in live cell % was found between HS-5 cells treated with cisplatin and GO-pretreated cells that were given cisplatin. Live cell % reduced from around 91% in GO-pretreated cells to around 26% in GO + cisplatin-treated cells. In HeLa cells, the number of apoptotic cells increased after pretreatment with GO and cisplatin treatment, while in U87 apoptotic cells, the numbers are similar to those achieved only by cisplatin treatment. Simultaneously, U87 viability decreased after GO pretreatment and cisplatin treatment The results mentioned above suggest that U87 cells may undergo a different process compared to HeLa cells when it comes to cell death. This could be due to the mutated *pten* gene found in glioblastoma U87 cells [36], which is responsible for regulating cell growth, metabolism, and survival through the PI3K pathway [37,38]. Unlike U87, HeLa cells do have the wild type of *pten* and *tp53*, which allows them to perform apoptosis by a caspase-dependent pathway. Based on other authors’ results, it can be assumed that due to the *pten* loss cells switch on an alternative mechanism, instead of apoptosis, premature senescence, which also inhibits proliferation. A previously mentioned mechanism was used to explain decreased cell activity in glioblastoma U87 cells exposed to ionizing radiation [36].

**Figure 9:**
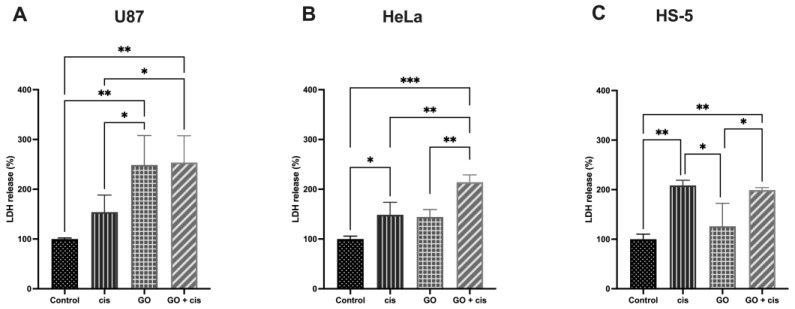
Comparison of the LDH release of U87 (**A**), HeLa (**B**), and HS-5 (**C**), showing cells pretreated with GO (GO), treated with cisplatin (cis), and pretreated with GO that were given cisplatin after GO treatment (GO + cis). Three asterisks (***) mean *p*-value ≤ 0.001, two asterisks (**) mean *p*-value ≤ 0.01 and one asterisk (*) means *p*-value ≤ 0.05. Abbreviations: cis, cells treated with cisplatin in a specific concentration for each line; and GO, cells pretreated with 1 μg/mL.

Repeated administration of GO was performed to limit GO use and its concentrations to be in safe, low concentrations that, as mentioned in the literature [13], do not affect cell viability, while potentially affecting cell membranes. Many researchers have shown that GO can cause membrane damage, and thus lactate dehydrogenase (LDH) release was measured [39,40]. We hypothesized that membrane damage and pore formation can result, and, in addition, the cisplatin can be taken into the cell by previously described mechanisms based on copper transporters, CTR1, CTR2, and passive diffusion [41,42]. LDH release assay results are presented in Figure 9. For U87, the highest LDH release was detected for cells pretreated with GO and cells that were given cisplatin following GO administration, around 248% and 253%, respectively. There were no significant differences in LDH release between both, but both were higher than in the control or in the GO-pretreated group. In HeLa cells, the highest LDH release was detected for the GO + cisplatin-treated cells at 214%. This was significantly higher than in any other group for the cell line. There was no significant difference in the LDH release between cisplatin-treated cells and GO-pretreated cells. The HS-5 analysis did not show any significant differences between cells treated with cisplatin and cells that were pretreated with GO and followed by cisplatin treatment. No significant change was found in LDH release between the control group and GO-pretreated cells. While a significant increase in LDH release compared to the control was detected in U87, in the case of other used cell lines, the LDH release levels tend to be high. Li et al. reported increased LDH levels in the bronchoalveolar lavage fluid of rats that were administered GO [25]. For increased U87, LDH release in GO-pretreated cells was the only one that resulted in decreased cell viability in opposition to the apoptosis assay result. Non-significant changes in LDH release, in GO-pretreated cells, could mean that GO caused nonlethal damage to the cell membrane.

**Figure 10:**
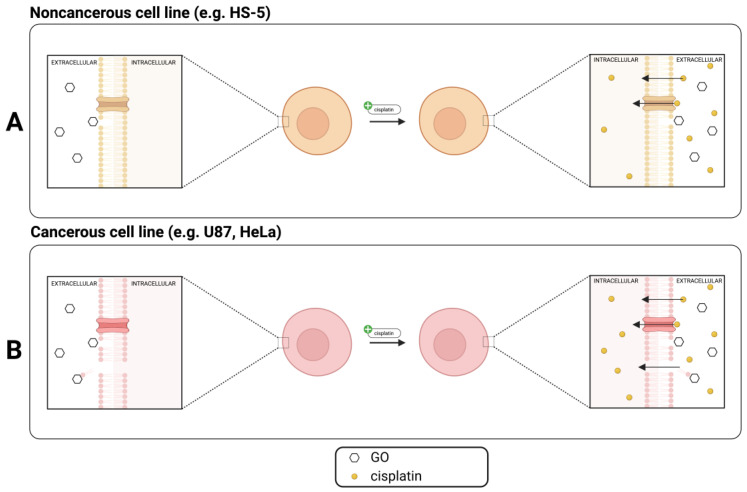
Proposed mechanism of GO enhanced cisplatin toxicity in U87 and HeLa cells. Created with BioRender.com.

What is important from a further application perspective is that we reported that GO-pretreated cells of cancerous U87 and HeLa cell lines responded more strongly to cisplatin treatment than those that were just cisplatin-treated, based on AlamarBlue and/or Annexin V/PI and/or cell morphology analysis, resulting in a significant decrease in cell viability. The above suggests that low-dose GO pretreatment followed by cisplatin administration can reduce cancerous cells’ viability while at the same time not causing additional harm to noncancerous HS-5 cells Figure 7C,D and Figure 8). This phenomenon can be based on the difference in membrane potential of cancerous and noncancerous cells [43] and GO’s electrostatic and hydrophobic properties [44]. Previous studies showed that other carbon-based nanoparticles—for example, graphene quantum dots—can enhance the toxic cisplatin effect in the HeLa cell line [45]. We propose a GO membrane damage and cisplatin uptake mechanism, which is presented in Figure 10. Our proposed mechanism is in line with the mechanism described by Sui et al. and Perini et al. [45,46] and our experimental data. GO interacts with noncancerous cells, causing minor damage to the lipid membrane. Damage is not enough to alter the integrity of the lipid bilayer and does not allow cisplatin to enter the cell in any way other than using membrane transporters or passive diffusion (Figure 10A). In cancerous cells, GO damages the membrane making pores and altering permeability, allowing additional cisplatin inflow into the cells, resulting in cell death. We suggest that nanopores, which are formed after GO administration, are not lethal to the cells and allow cisplatin (as presented in our paper) or other drugs to enter cells, enhancing therapeutic effects.

## 3. Materials and Methods

### 3.1. XPS Analysis

The XPS analyses were carried out in the Academic Centre for Materials and Nanotechnology, AGH University of Science and Technology, Cracow, Poland, using a PHI Versa Probe II Scanning XPS system (Physical Electronics, Inc., Chanhassen, MN, USA) with monochromatic Al Kα (1486.6 eV) X-rays focused to a 100 μm spot. The photoelectron take-off angle was 45°, and the pass energy in the analyzer was set to 117.50 eV for survey scans and 46.95 eV for obtaining high energy resolution spectra for the C 1s, O 1s, Si 2p, K 2p, and N 1s regions. A dual beam charge compensation with 7 eV Ar+ ions and 1 eV electrons was used to maintain a constant sample surface potential regardless of the sample conductivity. All XPS spectra were charge referenced to the unfunctionalized, saturated carbon (C-C) C1s peak at 285.0 eV. The operating pressure in the analytical chamber was less than 3 × 10^−9^ mbar. Deconvolution of spectra was carried out using PHI MultiPak software (v.9.9.3). Spectrum background was subtracted using the Shirley method.

### 3.2. Cell Cultures

In our research, we used the following cell lines obtained from American Type Culture Collection (Manassas, VA, USA): human glioblastoma U87 (ATCC^®^, Manassas, VA, USA, HTB-14™), human epithelioid cervix carcinoma HeLa (ATCC^®^ CCL-2™), and human bone marrow stromal HS-5 (ATCC^®^ CRL-11882™). Cells were cultured in 75 cm^3^ cell culture flasks using Dulbecco’s modified Eagle’s medium (DMEM), high glucose (Biological Industries, Beit Haemek, Israel) with the addition of 10% fetal bovine serum (FBS) (Life Technologies, Houston, TX, USA) and 1% penicillin and streptomycin (Life Technologies, Houston, TX, USA). Cells were grown in an INCOMED153 Memmert (GmbH & Co. KG, Schwabach, Germany) incubated at 37 °C, 5% CO_2_, and 70% humidity.

### 3.3. Preparation of the Graphene Oxide Dispersion

We obtained 0.4% graphene oxide from Advanced Graphene Products (Zielona Góra, Poland). GO was prepared from graphite using the modified Hummers method. GO had carbonyl groups, carboxyl groups, hydroxyl groups, and epoxy groups identified by the FLIR analysis [30].

### 3.4. Cell Pretreatment with GO

GO pretreatment, as the administration of low doses of GO to the cell lines, was performed to sensitize cells to further cisplatin treatment.

Each cell line was seeded at different vessels: for apoptosis assay, 1 × 10^4^ were seeded on 6-well plates; for viability and morphology, 1 × 10^4^ cells were seeded in 75 cm^3^ cell culture flasks. Each plate was divided into 2 equal parts—control and cells to be pretreated with 1 μg/mL GO. Accordingly, flasks that were designated to be given GO were chosen. The group pretreated with GO received DMEM containing 1 μg/mL GO, while the rest were treated with DMEM without any additives. After adding cell media, we incubated the vessels for 48 h. After incubation, the cell medium was replaced with a fresh cell medium (GO-treated cells were treated with DMEM containing 1 μg/mL GO, while non-GO-treated cells were given DMEM without any additives). The procedure was repeated 3 times.

### 3.5. Determination of Cisplatin Concentration for Further Treatment of U87, HeLa, and HS-5 Cells

To determine the concentration of the cisplatin for the further treatment of the cells, we applied 1 × 10^4^ cells per well of 96 well plates and then incubated them for 24 h in the incubator (T = 37 °C, 5% CO_2_, and humidity 70%). After 24 h, we replaced the medium with the medium containing cisplatin in the 9:1 ratio, which caused cisplatin concentrations to be 10-fold lower—500, 250, 125, 62.5, 31.25, 15.625, and 7.8125 μM. After 24 h of incubation, we added 11 μL of AlamarBlue to each well and incubated them for an additional 3 h. After incubation, fluorescence (570 nm for excitation and 600 nm for emission) was measured using an Infinite M200 plate reader (Tecan, Durham, NC, USA). The controls were cells that were not treated with cisplatin. The procedure was repeated for each cell line.

### 3.6. Cisplatin Treatment of Triple GO-Pretreated Cells

Cells for viability assay were trypsinized and seeded at 1 × 10^4^ per well on 96-well plates. A medium containing the designated cisplatin concentration was added to the cells and incubated for 24 h in the incubator (T = 37 °C, 5% CO_2_, and humidity 70%). After 24 h incubation with cisplatin, 96-well plates were used for viability tests, 6-well plates for the apoptosis assay, and 75 cm^3^ cell flasks for morphology analysis. For cell viability tests, 11 μL of AlamarBlue was added to each well and incubated for an additional 3 h. After incubation, fluorescence (570 nm for excitation and 600 nm for emission) was measured using an Infinite M200 plate reader (Tecan, Durham, NC, USA). Cells that were not treated with cisplatin were the controls. The procedure was repeated for each cell line.

### 3.7. Morphology Analysis

After cisplatin treatment, cells of each line were photographed using a Nikon Eclipse Ti (Nikon, Tokyo, Japan inverted light microscope at 10× magnification). The procedure was repeated for each cell line.

### 3.8. Apoptosis Assay

Apoptosis was assessed using an FITC Annexin V/Dead Cell Apoptosis Kit (Thermo Scientific, Waltham, MA, USA). U87, HeLa, and HS-5 cells were seeded at 1 × 10^4^ cells per well in 6-well plates. After 24 h, cells were treated with GO, as previously described. After three GO doses, cisplatin was added and incubated for 24 h. After incubation, cells were trypsinized, harvested, washed with cold PBS, and transferred to centrifuge tubes. Cells were centrifuged at 200× *g* for 5 min to form a pellet. The supernatant was discarded, and cells were suspended in 100 μL of Annexin binding buffer, followed by adding 5 μL of FITC Annexin V and 1 μL of propidium iodine. After that, cells were incubated in the dark at room temperature for 15 min, followed by adding 400 μL of Annexin binding buffer and gentle mixing. Cells were stored on ice until flow cytometry analysis. Cells were placed in a BD FACSCalibur (Becton Dickinson, San Jose, CA, USA) flow cytometer, and 10^4^ events were recorded per sample.

### 3.9. LDH Release Assay

To assess LDH release, cells pretreated with GO and the not treated control were seeded at 96 well plates at 10^4^ cells per well and incubated overnight. Half of the triple GO-treated cells and half of the control cells were treated with cisplatin and incubated for 24 h. Then, CytoScanTM LDH Cytotoxicity Assay kit (St. Louis, MO, USA) was used following the manufacturer’s protocol. Absorbance was measured at 490 nm and reference at 680 nm. Results were calculated as follows:

$$LDH\;release\;\left(\%\right)=\frac{100\%\times LDH\;\%\;change\;compared\;to\;control}{\%\;viability\;for\;group}$$

### 3.10. Statistical Analysis

The data obtained were analyzed by one-way variance analysis using GraphPad Prism 9 (GraphPad Software Inc., La Jolla, CA, USA). Differences between the groups were tested using the unpaired t-test and using variance analysis (ANOVA) supported by Benferroni’s post hoc test. All mean values are presented using standard deviation. Differences at *p* < 0.05 were considered significant.

## 4. Conclusions

The in vitro experiment showed that pretreatment of the U87 glioblastoma cell line and HeLa cervical cancer line with GO-sensitized cells and cisplatin treatment resulted in decreased cell viability compared to just cisplatin treatment. While reducing the viability of cancerous U87 and HeLa cell lines, GO pretreatment did not result in a decrease in noncancerous HS-5 cell viability after cisplatin treatment. It is worth noticing that each cell line responded differently. Presumably, this phenomenon is due to the “nanoscale damage” caused by GO to the cell lipid bilayer of cancerous and noncancerous cells. Taken together, these findings suggest that GO can be potentially used as a sensitizer in glioblastoma and cervical cancer to enhance the cisplatin effect, while not affecting healthy cells.

## Figures and Tables

**Table 1:** Surface composition (atomic %) determined by fitting XPS data.

	C			N	O		Si	K
Energy (eV)	285.0	287.2	288.9	399.8	531.6	532.8	102.3	293.2
**Groups**	C=C sp^2^ C-C sp3	C-O-C C-OH C-NH	C=O O-C-O	C-NH-	O=C	O-C O-Si	Silicone Siloxane	K^+^
**GO**	43.9	24.1	4.1	0.4	3.7	22.8	0.8	0.4

## Data Availability

The data presented in this study are available upon request to the corresponding author.

## Appendix A

**Table A1:** Flow cytometry apoptosis assay results.

Cell Line	Group	% Live	% Necrotic	% Late Apoptotic	% Early Apoptotic
**U87**	Control	79.43 ± 2.32	1.02 ± 0.36	4.21 ± 0.63	7.62 ± 1.21
	cis	55.15 ± 2.84	0.62 ± 0.25	9.05 ± 5.23	28.55 ± 1.58
	GO	81.51 ± 1.65	1.29 ± 0.49	4.18 ± 0.25	7.79 ± 0.60
	GO + cis	25.10 ± 14.57	0.67 ± 0.39	22.79 ± 14.16	45.45 ± 5.50
**HeLa**	Control	86.58 ± 2.73	1.59 ± 0.97	4.21 ± 0.63	7.62 ± 1.21
	cis	54.11 ± 11.26	8.62 ± 5.86	9.05 ± 5.23	28.55 ± 1.58
	GO	86.91 ± 0.52	1.13 ± 0.15	4.18 ± 0.25	7.79 ± 0.60
	GO + cis	25.10 ± 14.57	6.66 ± 0.86	22.79 ± 14.16	45.45 ± 5.50
**HS-5**	Control	90.32 ± 1.13	2.07 ± 0.72	1.67 ± 0.11	5.93 ± 1.97
	cis	22.51 ± 2.53	3.05 ± 1.44	42.63 ± 2.70	31.81 ± 1.32
	GO	90.86 ± 0.30	2.01 ± 0.28	2.00 ± 0.32	5.12 ± 0.78
	GO + cis	26.20 ± 5.25	11.73 ± 13.38	34.55 ± 12.08	27.52 ± 6.57
